# Optimizing the co-digestion supply chain of sewage sludge and food waste by the demand oriented biogas supplying mechanism

**DOI:** 10.1177/0734242X20953491

**Published:** 2020-09-10

**Authors:** Yiyun Liu, Tao Huang, Daoping Peng, Jingjing Huang, Claudia Maurer, Martin Kranert

**Affiliations:** 1 Faculty of Geosciences and Environmental Engineering, Southwest Jiaotong University, China; 2 University of Stuttgart, Institute for Sanitary Engineering, Water Quality and Solid Waste Management, Germany

**Keywords:** Co-digestion, demand oriented, supply chain, optimization, system dynamics

## Abstract

Co-digestion of sewage sludge with food waste is a beneficial pathway for sewage plants to enhance their biogas yield. This paper employs hybrid programming with system dynamics simulation to optimize such a co-digestion system from the perspective of demand-oriented biogas supply chain, thus to improve the efficiency of the biogas utilization. The optimum operational parameters of the co-digestion system are derived from the simulation model. It is demonstrated that the demand-oriented biogas supply mechanism can be effectively driven under market-oriented incentive policy. For better compensation of the external cost to assist the operations of the co-digestion supply chain, it is suggested that the substrate collection and transportation subsidy should be combined with the renewables portfolio standard to be implemented as the optimum incentives. The limitations of the study are discussed to lay the foundation for future improvements.

## Introduction

With the increase of newly built sewage plants, large amounts of excess sludge have been produced (Prabhu and Mutnuri, 2016). Anaerobic digestion (AD) has been regarded as a promising method for sludge stabilization, and the produced biogas can be used for electricity production as a renewable energy source (Glivin et al., 2016; Luo et al., 2019). However, the relatively low organic content of excess sludge leads to a low biogas production rate, which means it is not beneficial for implementing in sewage plants (Yuan and Zhu, 2016). Some researchers have tried the co-digestion of municipal food waste and sewage sludge to increase the organic load rate and stability of the AD system, thus enhancing the biogas production rate to improve the economic benefits from power generation (Mehariya et al., 2018; Nghiem et al., 2017). The sewage plant can make full use of the digester’s capacity through the co-digestion to treat the self-produced excess sludge and municipal food waste simultaneously, so as to achieve a win–win–win of economic, environmental and social benefits (Glivin et al., 2020; Park et al., 2016).

Most co-digestion systems are operated based on the continuous stirred tank reactor (CSTR) system, in which biogas can only be supplied to generate electricity at a fixed output rate so that the electricity output is difficult to flexibly regulate in accordance with the actual demand of the sewage treatment plant, which leads to low biogas utilization efficiency (Lensch et al., 2016; Mao et al., 2015). The ‘demand-oriented biogas supply’ (DO) proposed by Germany provides an effective solution to this deficiency of the traditional co-digestion system (Szarka et al., 2013). Through adopting the DO mechanism, the electricity demand of the sewage plant can be satisfied based on the advantage of biomass energy being easy to artificially control compared with other renewable energy sources (Budzianowski, 2016; Häring et al., 2017). By regulating the biogas utilization scheme from the expanded biogas storage tank, or adjusting the AD reaction process, the biogas can be supplied for electricity generation in real time and in certain amounts in response to the daily electricity demand (Hahn et al., 2014b; Lauer and Thrän, 2018).

However, if demand-oriented biogas production is introduced into a sewage plant’s co-digestion system, huge investments and operation costs from newly built digesters and security equipment will be necessary for the sewage plant, since the plant is required to expand the biogas production capacity during the peak hours of electricity consumption (Barchmann et al., 2016; Hahn et al., 2014b). Secondly, the co-digestion system is a supply chain involving raw material collection and transportation, AD reaction, biogas storage and purification, power generation and utilization (Balussou et al., 2012; Smith et al., 2017). When the DO mechanism is employed, it inevitable that the supply chain operations will be disturbed (Bekkering et al., 2013).

In this context, this study intends to introduce the DO to optimize the co-digestion supply chain. The optimal operation conditions of the biogas production and power generation system under the DO mechanism are derived from a hybrid mixed integer linear programming (MILP) and system dynamics method, and the corresponding policy incentives are designed to assist the operation of the DO mechanism. It is expected that this research could provide some theoretical basis for recycling of secondary pollutants in municipal sewage plants, as well as enhancing their bio-energy utilization efficiency, thus finally improving their energy resource structure.

## Literature review

Researches in the field of co-digestion of sewage sludge and food waste in sewage plants have mainly been carried to explore its technical feasibility. For example, Mattioli et al. (2017) took the co-digestion technology of a sewage plant in Rovereto, Italy, as a case example; the results showed that the organic loading rate (OLR) could be doubled without any process difficulties, and the increased biogas yield could satisfy 85% of the plant’s energy demand. Similar conclusions have also been drawn by Björn et al. (2017), who proved that co-digestion with food waste has the potential to increase biogas production by approximately four times. Similar techno-economic studies can also be found in Glivin et al. (2018, 2019b), Lamidi et al., 2019 and Walekhwa et al. (2014). Apart from economic feasibility, researchers have also paid attention to the co-digestion systems’ environmental performance; for instance, Edwards et al. (2017) evaluated the co-digestion system and sole digestion by the life cycle assessment (LCA) method; the results showed that co-digestion has the least environmental impact across all categories, excluding human toxicity, and increasing specific biogas production shows a global warming potential decrease of at least 2.5%. Similarly, Chiu et al. (2016) compared the current incineration treatment method and the proposed co-digestion for sewage sludge and food waste in Macau by LCA; the results demonstrated that the proposed co-digestion scenario could improve the performance in human health, ecosystems and energy production by 36%, 13% and 61%, respectively.

In addition to feasibility studies, researches have also studied how to improve the biogas production efficiency of the co-digestion system. Most researchers optimized the co-digestion’s reaction process by regulating the AD reaction parameters to increase the biogas and methane production rate. In this field, Koch et al. (2015) optimized the co-digestion process based on the mixture ratio of food waste and sludge; it was shown that the methane yield rose linearly with the increased contribution of food waste due to its higher methane potential and methane production rate. Gou et al. (2014) investigated the effects of the OLR on the system performance; it was found that a low OLR (<5 gVSL^−1^d^−1^) could be used for the boosting the CH_4_ yield and volatile solids (VS) removal efficiency. Meanwhile, Fitamo et al. (2016) focused on the parameter of hydraulic retention time (HRT), and their results revealed that the biogas production improved significantly in line with decreasing HRT, with the best performance was achieved at 15 days HRT with stable process parameters. Similar works have also obtained by Ratanatamskul et al. (2014), but different from the Fitamo et al. (2016), their results indicate that co-digestion should be combined with sufficient operating HRT in terms of the enhancement of methane concentration. Apart from the AD process parameters, some researchers also optimized the biogas production efficiency by introducing the pre-treatment method; for instance, Zhang et al. (2016) proved that microwave pre-treatment is valid for enhancing the biogas and methane yield. Yin et al. (2016) used fungal mash rich in hydrolytic enzymes for the pre-treatment of food waste and sludge; the bio-methane yield of mixed waste pre-treated with fungal mash was found to be 2.5 times higher than the scenario without pre-treatment. In addition, some researches started trials for enhancing the biogas production by adding other co-substrates with the food waste and sludge; for example, Kim and Kang (2015) added microalgal biomass as the co-substrate and Maragkaki et al. (2018) adopted cheese whey and olive mill wastewater; the methane and biogas production volumes after further co-digestion were increased by 72% and 170%, respectively; Quiroga et al. (2014) analysed the combined method of ultrasound pre-treatment and co-digestion of cattle manure with food waste and sludge; the results showed that the combined method allows the co-digestion system to operate at lower HRT while achieving higher volumetric methane yields.

The above-mentioned studies, although demonstrating the feasibility of the co-digestion of sewage sludge and food waste, only focused on how to increase the biogas production in order to maximize the conversion rate of the embodied energy from biomass; no research has considered the convenience and flexibility of biogas utilization. Secondly, most of the studies paid attention solely to the objective of the sewage plant, but ignored the overall disturbance to the supply chain system after the introduction of the co-digestion system. In order to make up for the deficiency of the existing research, this research introduces DO to improve the utilization efficiency of the co-digestion system, then further re-designs the system from the supply chain perspective; the optimal policy incentives are finally proposed to assist the operation of the DO mechanism.

## Modelling method

### System description and modelling hypothesis

**Figure 1.**
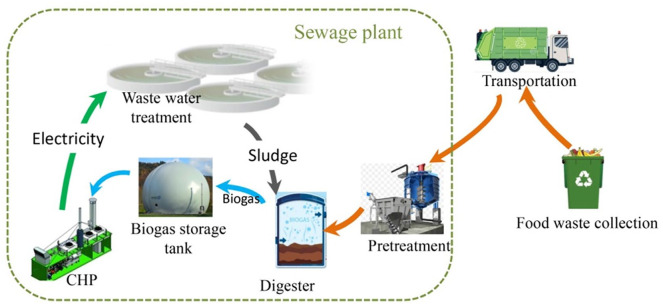
The proposed co-digestion conceptual system. CHP: combined heat and power.

The co-digestion system is constructed from the supply chain perspective, as shown in Figure 1. Food waste is intended to be collected and transported to the sewage plant by the agent. After pre-treatment, it is co-digested for biogas production by mixing with sludge from the secondary sedimentation tank. The biogas is stored in the installed storage tank and purified for methane extraction. Electricity is further generated through combined heat and power units (CHPUs) for self-use in the sewage plant.

For investigating the economic feasibility of the co-digestion system under the DO mechanism, hybrid programming with the system dynamics simulation method is proposed. MILP is constructed to obtain the optimal electricity production timetable and combined heat and power (CHP) operating conditions during one typical day under the DO mechanism. Based on the optimal electricity demands, the operations of the biogas production system can be simulated by system dynamics, for which the net present value (NPV) of the co-digestion system can be calculated. Finally, incentive instruments for the co-digestion system under the DO mechanism are proposed, with accompanying perspectives from managerial experiences.

In order to define the system boundary, some necessary assumptions are proposed as follows.

- The daily electricity demand during a typical day was selected as the representative parameter for modelling. Possible fluctuations from seasonal variations and the expansion of AD capacities were neglected.
- The daily property parameters of sewage sludge and food waste after pre-treatment are stable and no obvious fluctuations happen.
- The revenue from biogas slurry and residues after the fertilizer process are about the same as the cost of fertilizer production; so, the influence of the fertilizer process on the economic benefits of the system can be ignored after the introduction of the DO mechanism. The treatment process of biogas slurry and residues are not considered in the system boundary of this study.
- DO is mainly based on biogas storage regulation, and extra digesters are introduced to supply biogas in a flexible way for compensating the electricity demand during peak load periods. It is further assumed that the technical feasibilities can be guaranteed in this study.

### Mixed integer linear programming

The revenue of the demand-oriented biogas-to-electricity system is measured by the revenue regenerated from purchased electricity minus the start-up cost and operational cost of CHPUs. The objective function is set based on maximizing the net revenue, which is given as follows

(1) $$\begin{array}{l} max\;Revenue=P_{E}\cdot \sum_{t=0}^{23}min\;(D_{t},\sum_{i=1}^{n}E_{i}\cdot \;\;U_{it})\\ -\sum_{t=0}^{23}\sum_{i=1}^{n}C_{sta}\cdot \;\;U_{it}\cdot \;\;\left(1-U_{i,t-1}\right)\\ -\sum_{t=0}^{23}\sum_{i=1}^{n}C_{op}\cdot \;\;U_{it}\cdot E_{i} \end{array}$$

Supposing that a certain proportion of the sewage plant’s power demand must be satisfied by power generated from the co-digestion of sludge and food waste, the corresponding constraints are given as follows

(2) $$\sum_{t=0}^{23}\sum_{i=1}^{n}E_{i}\cdot U_{it}\geq \alpha \cdot \sum_{t=0}^{23}D_{t}$$

(3) $$U_{it}=\left\{0,1\right\}$$

where $P_{E}$ is the electricity purchase price for the sewage plant, $D_{t}$ is the daily electricity demand of the sewage plant in *t* hour, where *t* = 0,1,. . .., 23, $E_{i}$ is the electricity capacity of the *i*th turbine generator, where *i* = 1,2,. . ., *n*, $U_{it}$ is the 0–1 variable, where $U_{it}$=1 indicates that in *t* hour the *i*th turbine generator is switched on; otherwise, it is switched off, $C_{sta}$ is the unit start-up and shutdown cost and $C_{op}$ is the unit CHPU operation cost.

### System dynamics

**Figure 2.**
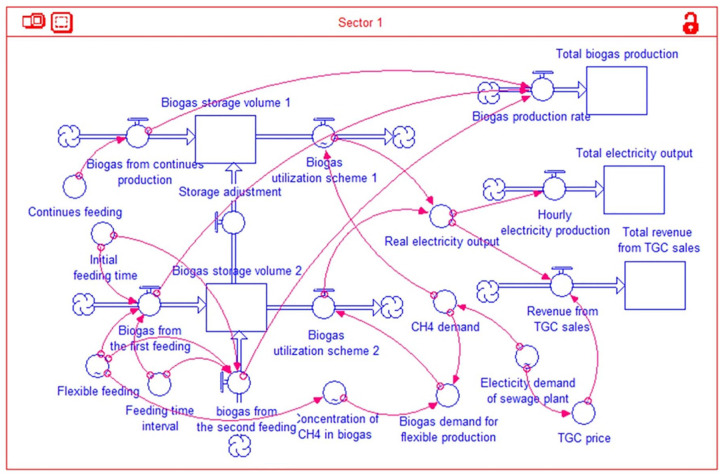
Stock-flow diagram of the SD model. TGC: tradable green certificate; SD: system dynamics.

System dynamics is beneficial for simulating the time-dependent variations of system behaviour (Forrester, 1994). STELLA software is adopted to build a stock-flow diagram for quantifying the interrelations among main variables within the DO system in the sewage plant, shown in Figure 2.

Quantitative relationships among variables are displayed in the Appendix. The main mathematical equations for predicting biogas production under the flexible operating mode (seen in Equations (4) and (5)) are based on the modified first-order kinetics model. For detailed explanations of this equation, refer to Liu et al. (2020)

(4) $$V\left(t\right)=V_{0}\left(t_{i}\right)+V_{t}^{i}$$

(5) $$V_{t}^{i}=v_{max}^{i}(OLR)\cdot (1-exp\left(-k^{i}(OLR)\cdot (t-t_{i})\right))$$

where V(t) is the accumulated biogas production (mL biogas L^−1^ digester) in feeding time *t* (min) from the arbitrary *i*th to (*i*+1)th feeding, $V_{0}\left(t_{i}\right)$ is the accumulated biogas production volume from the first feeding to the *i*th feeding, which belongs to a measured parameter, $V_{t}^{i}$ is the biogas production generated specifically from the *i*th feeding, $v_{max}^{i}(OLR)$ is the final specific methane produced (mL biogas L^−1^ digester) at the end of the assay, which is the function of the organic loadings in the digester and $k^{i}(OLR)$ is the first-order decay constant (1 min^−1^), which is also regarded as the function of the OLR.

Meanwhile, for the continuous biogas production mode, the equation for predicting the biogas production can be expressed as follows (O’Shea et al., 2016)

(6) $$V_{biogas}^{Con}=\nu_{biogas}\cdot V_{reactor}$$

where $\nu_{biogas}$ is the biogas production per unit volume of reactor (m^3^ gas m^−3^ reactor) and $V_{reactor}$ is the reactor volume (m^3^).

After regulating the biogas production, the biogas can be exported for electricity generation, in which the electricity output can be calculated by using the following equation (O’Shea et al., 2016)

(7) $$kW_{e}=BU_{biogas}\cdot MC\cdot \eta_{e}\cdot 10.49$$

where $BU_{biogas}$ is the biogas utilization scheme for continuous or flexible biogas production, MC is the methane content in the biogas, $\eta_{e}$ is the electrical efficiency of the CHPUs and ‘10.49’ is the energy content of methane (kWh^th^ Nm_3_CH_4–1_).

### Economic benefits accounting

**Figure 3.**
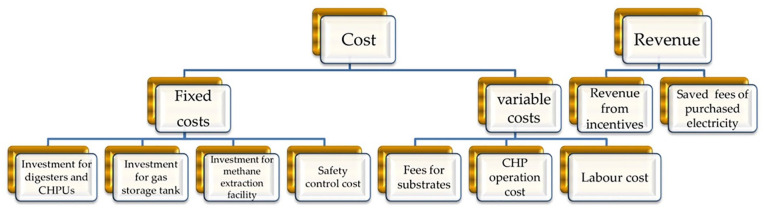
Classifications of cost and revenue involved in economic accounting. CHPU: combined heat and power unit; CHP: combined heat and power.

The composition of revenues and costs are shown as Figure 3, in which the costs are mainly divided into the fixed costs and variable costs. The former consists of one-off investments on the digesters, CHPUs and biogas storage tanks, and safety control systems. The latter contains fees for substrates, CHP operation costs and labour costs. The revenues of sewage plant under the DO mechanism are composed of the savings from purchasing the electricity as well as revenue from incentives (i.e. governmental subsidies), in which the former indicates the cost subtracted by the electricity from co-digestion. In this study, the NPV is selected as the economic indicator of the sewage plant, given in Equation (7) (Kim et al., 1986). The larger the NPV, the higher the economic benefit that can be achieved

(8) $$NPV=-CF_{0}+\sum_{y=1}^{k}\frac{CF_{y}}{{\left(1+R\right)}^{y}}$$

where *CF*_0_ is the initial investment for demand-oriented supply transformation, *CF_y_* is the cash flow in year *y*, where *y* = 1, 2, ···, *k*, and *R* is the interest rate.

## Data acquisition and model validation

In this study, a sewage plant in Chengdu, Sichuan Province, is taken as a typical case example for data acquisition and model verification. The sewage plant was constructed with the daily treatment capacity of 300,000 m^3^ waste water and produced sludge volume of 400 t day^−1^. After the on-site drying and dehydration processes, the residual sludge is transported to the landfill for final disposal. The digesters have already been equipped for excess sludge AD, but have not been put into operation. Parameters related to the sludge are derived by field investigation, shown in Table 1. Data for input parameters are mainly derived from similar co-digestion projects, shown in Table 2.

**Table 1.** Characteristics of feeding substrates.

	Water content (%)	TS (%)	VS (% dry weight)	COD (g COD L^−1^)	Carbohydrate (%)	Fat (%)	Protein (%)
**FW**	81.7	17.2	93.03	64.64	42.05	16.38	19.52
**Sludge**	99	0.84	69.1	8.77	–	–	–

TS: total solids; VS: volatile solids; COD: chemical oxygen demand; FW: food waste.

**Table 2.** Measurement of the model input parameters.

Input parameter	Value	Measurement
**Volume of digester**	2000 m ^3^ digester^−1^	From the field investigation
**CHP electrical efficiency**	40%	O’Shea et al. (2016)
**Investment for unit volume of digester**	4175.1	Grim et al. (2015)
**Unit food waste collection and transportation cost**	50 Yuan t^−1^ FW	Field investigation from the Chengdu food waste recycling plant
**Economic life span** **Construction**	20 years	Hahn et al. (2014a)
**Interest rate**	6%	Hahn et al. (2014a)
**Coefficient of maintenance and controlling cost for flexible biogas production**	2% of the initial investment	Lauer et al. (2017)
**Unit start-up cost of CHPU**	48 Yuan start-up^−1^	Grim et al. (2015)
**Labour costs**	50 Yuan person^−1^.day	From field investigation.
**Electricity price for sewage plant**	0.75 Yuan kWh^−1^	From field investigation
**Electricity demand of sewage plant in a typical day**	Lookup function, seen in the Appendix	From the operation report of sewage plant
**Governmental subsidy for renewable electricity**	0.25 Yuan kWh^−1^	Renewable electricity quota and assessment methods
**Energy content of 1 m** ^3^ **methane**	37.78 MJ/(3.6 MJ kWhth^−1^) = 10.49 kWhth	O’Shea et al. (2016)
**Self-consumption rate of electricity under DO mode**	13%	According to the calculation by Lauer et al. (2017), the self-consumption rate of electricity for methane safety system and expanded plant facilities is about 13% of the total electricity generation from DO system.

CHP: combined heat and power; CHPU: combined heat and power unit; DO: demand-oriented biogas supply; FW: food waste.

To validate the proposed simulation model, the simulated results of electricity generation are compared with the operation data of existing biogas plants. Through co-digestion, the simulated daily electricity output can reach 11,280 kW day^−1^ without the implementation of DO, which is equivalent to 318.15 kW t^−1^ VS after transforming into organic matter-based electricity production for the sake of comparing with other data of biogas plants. According to the data of co-digestion plants around the world summarized by Nghiem et al. (2017) in their review work, the actual electricity generation ranges from 28.81 kWh t^−1^ VS (Camposampiero–Italy) to 498.74 kWh t^−1^ VS (East Bay MUD–USA) by using the co-digested substrates food waste and excess sludge. Thus, the simulated results can be regarded to be within the reasonable range, which could further prove the feasibility of the proposed simulation model.

## Results and discussion

### Electricity production

**Figure 4.**
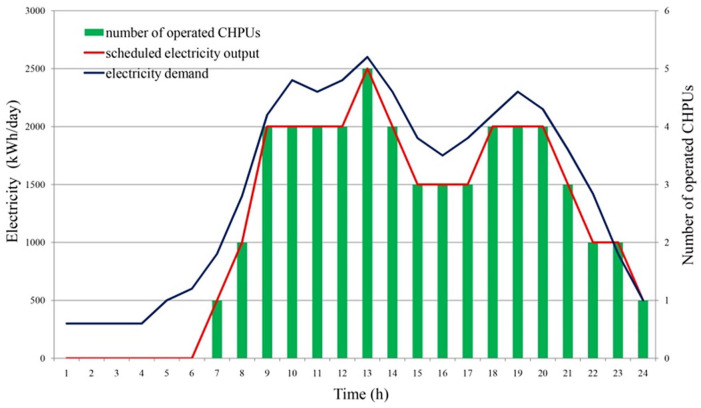
Determined electricity production schedule based on the mixed integer linear programming model. CHPU: combined heat and power unit.

Figure 4 shows the solutions of the MILP model, in which the CHPU operation schemes and the hourly biogas utilization timetables are determined. CHPUs operate from 7 am in fully flexible mode and during the peak load period (9 am to 2 pm and 6 pm to 8 pm), where more CHPUs need to be operated for centralized biogas production.

### NPV of biogas plant

**Figure 5.**
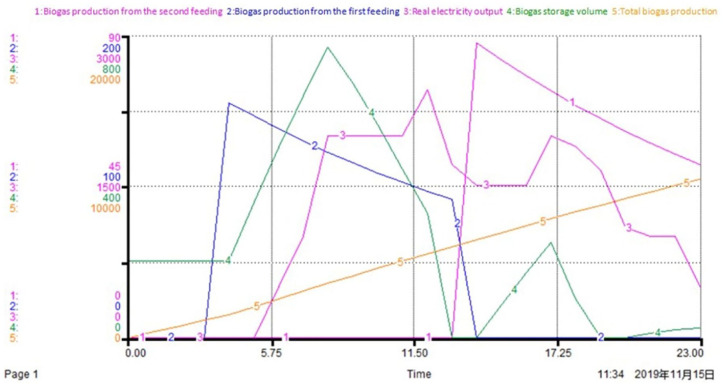
SD simulation results of some key operating parameters. SD: system dynamics.

System dynamics is employed to simulate the operation of the DO mechanism within 24 hours, and the variation trends of the key operating parameters of the system (such as the biogas production rate, actual electricity output, biogas storage capacity, total biogas production volume, etc.) are shown in Figure 5.

**Figure 6.**
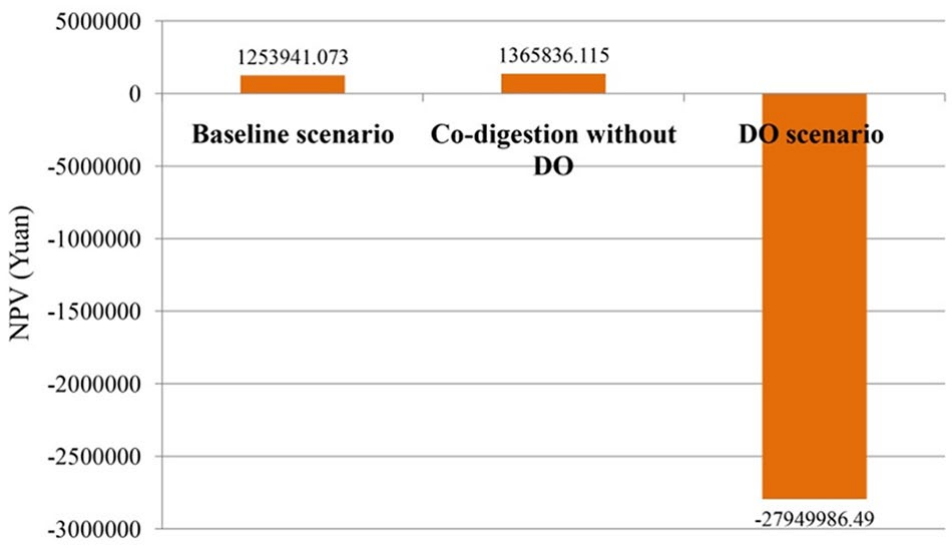
Net present value (NPV) accounting results of scenarios based on a governmental electricity subsidy. DO: demand-oriented biogas supply.

Based on the simulation results, the NPVs of the co-digestion system before and after the implementation of the DO mechanism are calculated, as shown in Figure 6. The solely AD sewage sludge is set as the benchmark scenario for demonstrating the economic benefits of the co-digestion. According to the accounting results, the NPV is greater than 0 when the co-digestion is introduced into the sewage treatment plant, indicating that certain economic benefits can be achieved with the co-digestion system’s introduction. The daily biogas production volume can reach 4766 m^3^ under the co-digestion system, which is able to produce 11,280 kW electricity per day, resulting in 31.8% of total electricity of the sewage treatment plant being covered by the renewable energy source, and 197 t day^−1^ food waste can be co-treated simultaneously. Although the biogas yield of the co-digestion system increased by seven times compared with the benchmark scenario, its NPV only increased slightly, which shows that significant investment and operating costs (mainly food waste collection and transportation costs) will be introduced with the co-digestion system, thus positively affecting the economic benefits of the system.

After the implementation of the DO mechanism, higher electricity output can be achieved during the peak load period and the operation of the CHPUs during the base load period can be cut down, and thus the biogas utilization efficiency can be improved and more bio-energy electricity can be supplied for the sewage plant’s power generation system as well. Under the DO mechanism, the biogas production volume of the co-digestion system reaches 10,434 m^3^ per day, equivalent to 66% of the sewage plant’s electricity demand; at the same time, 538 t day^−1^ of food waste can be recycled. However, the NPV of the DO mechanism is negative, which indicates that a heavy burden of investments and operating costs of the sewage treatment plant will be brought by the system, which the increased revenue from biogas increments cannot cover, resulting in significant improvements in environmental performance, but a decline in economic benefits. On the other hand, the current uniform levels of current government subsidies for renewable electricity are unable to effectively encourage the DO to compensate for the external cost of the system; instead, heavy financial burdens will be imposed on the government.

### Effects of policy instruments

In order to monetize the positive externalities of bio-energy-based electricity utilization in sewage plants, this study adopted the renewables portfolio standard (RPS) as a typical market-oriented incentive policy to analyse its auxiliary effect on the DO. Under the RPS policy, the government will set quotas to ensure that a certain proportion of electricity generated is from the renewable source for each power plant, so that the electricity generated from AD in the sewage plant can be counted into the trading system and the plant can obtain a certain number of green certificates (Sun and Nie, 2015; Zhang et al., 2017). In the tradable green certificate (TGC) market, sewage plants can profit from selling TGCs to power plants that have a demand for green certificates (usually thermal power plants, etc.) (Anguelov and Dooley, 2019). The TGC price is determined by the supply and demand of TGCs in the market; on this basis, sewage plants can sell more TGCs at higher TGC prices during the period of peak electricity demand, which could form effective incentives for compensating for the external cost of the DO mechanism.

This study assumes that all the electricity generated from co-digestion can be converted into green certificates and traded in the market, and the transaction price adopts the average value of the predicted TGC price over 20 years from Zhang et al. (2017). It is further assumed that the variation of TGC price during one day is proportional to the TGC demand.

**Figure 7.**
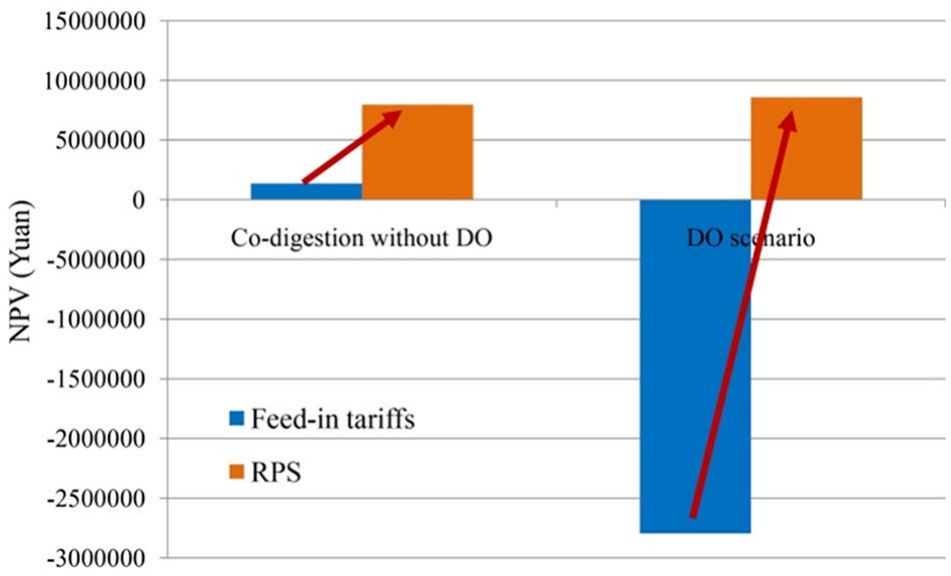
Improvement effects of co-digestion scenarios under the renewables portfolio standard (RPS) scheme. NPV: net present value; DO: demand-oriented biogas supply.

Figure 7 shows the enhancement effects of the NPV under the RPS policy compared with the traditional government subsidy. After the implementation of the RPS policy, the NPV under the DO mechanism increased significantly to a value greater than 0, while the enhancement effect of the traditional co-digestion scenario was far less than the DO, which demonstrated the incentive effects of the RPS policy on the DO mechanism. However, the NPV of the DO mechanism after implementation of the RPS policy is still only slightly higher than that of the traditional co-digestion scenario, indicating that although part of the external costs of the DO mechanism can be compensated for by the RPS policy, the effectiveness on the economic benefits is still limited; thus, the impetus for the DO mechanism to be implemented by the sewage plant is lacking.

In order to determine the main factors restricting the NPV of the DO mechanism, sensitivity analysis was further conducted by varying the uncertain input parameters of the co-digestion ceteris paribus within a 10% interval (Hahn et al., 2014a). The flexibility (defined as the ratio of biogas compensating for peak load electricity demand phase to the base load continuous biogas production), co-digestion facilities investments, feeding OLR of substrates, real biogas production amount and labour salary are selected as the key parameters for representing the possible uncertain factors that have direct impacts on the economic benefits of the proposed system (Laperrière et al., 2017). The extent of the variation of the output (NPV of system) indicates the risk potential of each parameter on the economic benefits of the sewage plant.

**Figure 8.**
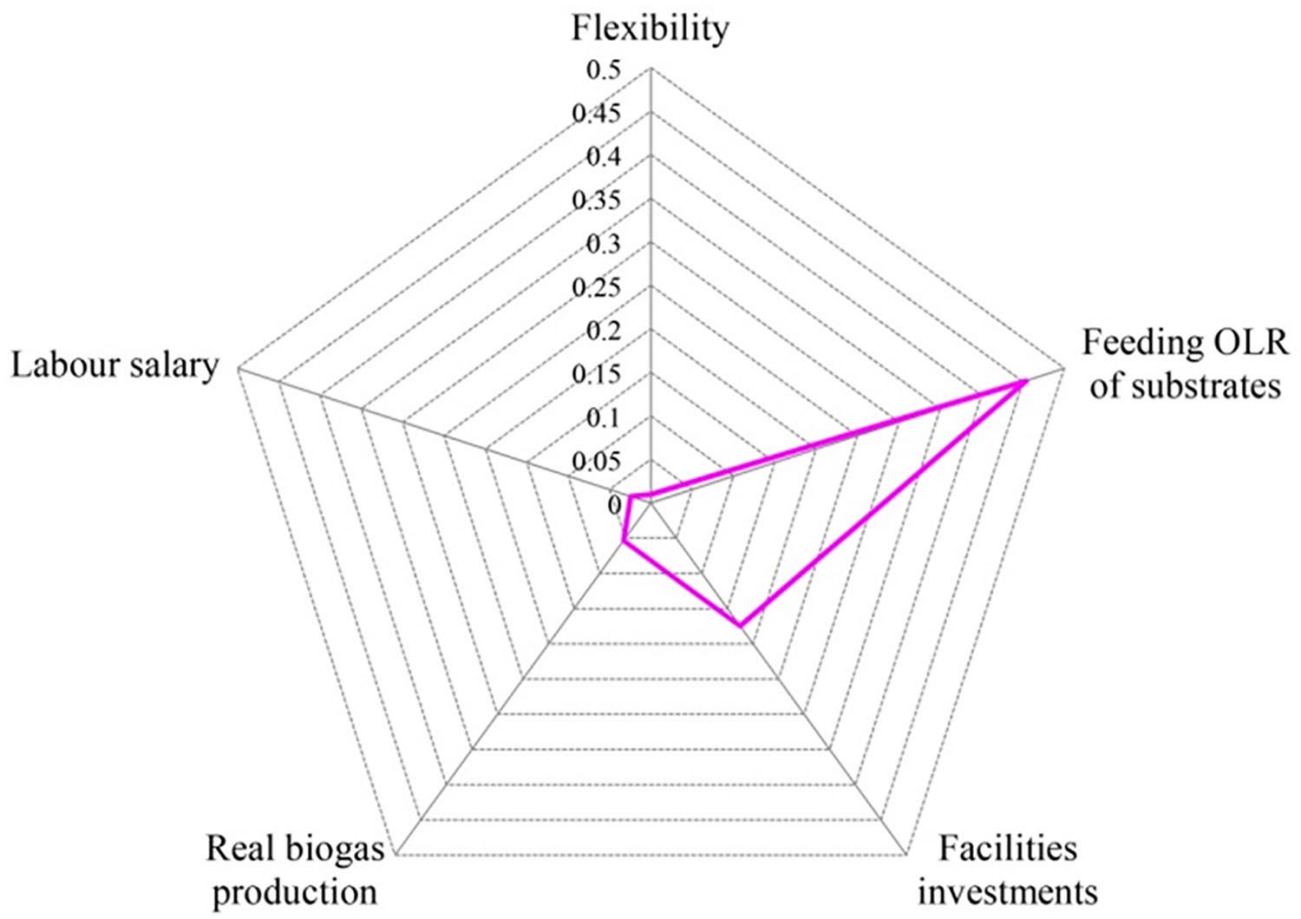
Results of sensitivity analysis. OLR: organic loading rate.

The results of sensitivity analysis are shown in Figure 8. It is illustrated that the feeding OLR is the critical factor, which may lead to a significant decline of benefits due to large collection and transportation costs. This is consistent with the conclusions of Hahn et al. (2014a) and Skovsgaard et al. (2017), who identified that the substrates cost was the key factor in influencing the economic profits of the biogas system. On the other hand, such implication confirms the results that the decrease of economic benefits caused by the higher substrates demand may make it infeasible for the proposed system to operate.

**Figure 9.**
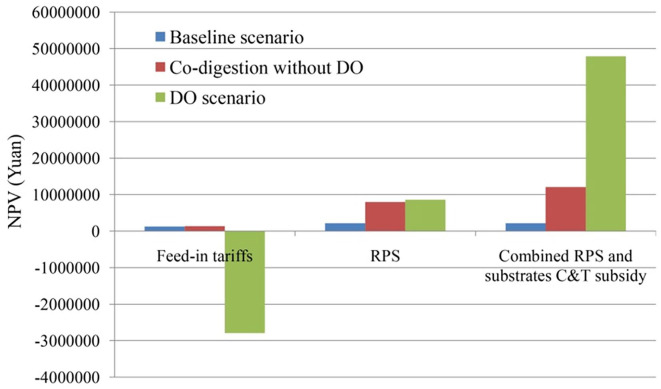
Net present value (NPV) of scenarios under the combined renewables portfolio standard (RPS) and governmental collection and transportation subsidy (C&T) subsidy. DO: demand-oriented biogas supply.

Therefore, this paper further attempts to introduce the governmental substrates collection and transportation subsidy (C&T) policy combined with the RPS to assist DO operations. Suppose that the government gives a certain subsidy to the sewage plant based on the collection and transportation volume of food waste. According to the former calculation results of the NPV under the electricity subsidy and RPS policy, the C&T subsidy standard is assigned as 5 Yuan t^−1^ food waste. The results are shown in Figure 9.

According to the accounting results, the NPV of the system under the DO mechanism with the C&T subsidy is significantly higher than that of the co-digestion without DO implementation and benchmark scenarios. This indicates that the substrate C&T subsidy can effectively incentivize the operations of the DO mechanism to achieving a win–win for both economic and environmental performance. In addition, the governmental expenditure for the C&T subsidy per day is 2689.95 Yuan day^−1^, which is much lower than that under the electricity subsidy with 6699.49 Yuan day^−1^. Therefore, it is suggested that the combined policy instrument of market-oriented RPS and government C&T subsidy can be adopted to assist the DO mechanism when it is applied to sewage plants in the future. It should be noted that this study only selects the situation in which the government sets the C&T subsidy to a standard of 5 Yuan t^−1^ as a calculation example, but the determination process of C&T subsidy standards needs to be further considered by combining the specific food waste collection and transportation mode and the possible interactions among the stakeholders involved.

## Discussion

This study optimized the co-digestion of the sewage sludge and food waste system from the supply chain perspective, where the results proved the optimization effects of introducing the DO. However, implementation of the DO by the co-digestion of excess sludge and municipal food waste has no established engineering practices, so it still has some uncertainties for real application. Commonly used substrates for the DO are agricultural wastes, including crop straw and livestock manure. Example studies (Barchmann et al., 2016; Feng et al., 2018; Mauky et al., 2015) used cow manure and corn silage as substrates for AD to produce biogas on demand. Laperrière et al. (2017) further used corn silage, carrots and glycerol; Ahmed et al. (2017) adopted sugar beet silage and grass silage as substrates for co-digestion. These studies confirmed the suitability of selected agricultural wastes for using as regulatory substrates to regulate the biogas supply. This can be attributed to the fast degradation rate and biogas production rate after feeding, due to a composition of highly water-soluble carbohydrates (Glivin et al., 2019a). Food waste is also a type of substrate rich in starch, polysaccharide, fatty acids, proteins, dietary fibre and other biodegradable components, which could be decomposed and utilized easily by the microbes after feeding; thus, it could be regarded as a substrate with great potential for realizing the DO mechanism (Wang et al., 2014; Zhang et al., 2007). However, the sole AD of food waste can easily lead to acid inhibition (Meng et al., 2015). Co-digestion with sewage sludge is a valid way for the AD of food waste, since sewage sludge is able to provide the alkalinity and micronutrients required for the AD reaction (Nghiem et al., 2017). Some researchers have already shown that if the co-digestion of food waste and sludge is implemented, abrupt increments of the feeding OLR can be adopted without negative impacts on the AD reaction process performance (Liu et al., 2020). On the other hand, the key for implementation of the DO is a valid biogas production process control system (Mauky et al., 2016). Existing researches have explored the applications of the control system, such as proportional–integral–derivative (PID) control, fuzzy control, the artificial neural network, etc., for monitoring the demand-oriented operations (Batstone et al., 2002; Gaida et al., 2011; Haugen et al., 2013; Waewsak et al., 2010). Results show that the above-mentioned controlling models are useful for regulating the AD process and ensuring the stability of the system, which ensures the applicability of the DO by the co-digestion of food waste and sludge.

In addition, this study designed the combined governmental C&T and RPS incentive policy and verified its effectiveness for the DO; however, the related calculations are based on some simplifications and hypotheses. In fact, the allocation of renewable energy quotas for each power plant under the RPS policy and the formation of TGC prices are quite complex processes, which are determined by the interactions among thermal power plants, renewable power producers, government and electricity users, and restricted by the capacity and technical level of renewable energy power generation in this region (Zhao et al., 2018). Further researches should predict the effectiveness of RPS based on the possible behaviour interactions of involved stakeholders from the long-term perspective and select case examples within different regions. With regard to the enactment of substrate C&T subsidies, further researches should take into account the dispersion characteristics of food waste, and comprehensively analyse the formulation of standards for the C&T subsidy under different collection and transportation modes, for example centralized collection by sewage plants from large catering enterprises, third-party collection and transportation enterprises, purchase from other food waste recycling enterprises and so on, in order to further enrich and improve the proposed incentive instruments.

## Conclusion

This paper employs a hybrid programming with system dynamics simulation to investigate the economic feasibility of the DO by co-digestion of sewage sludge and food waste from the supply chain perspective. Firstly, the optimal electricity production timetable under the DO mechanism is solved by the MILP model; on this basis, the system dynamics model is established for simulating the operation of the co-digestion system. Based on the NPV accounting results, it is demonstrated that the co-digestion of sewage sludge and food waste is a beneficial method for sewage plants using bio-energy recovery. However, if the DO mechanism is introduced, the economic returns are not able to cover the related costs under the current governmental subsidy policy. The combined RPS and substrate C&T are identified as the optimal incentive instruments for the DO’s future implementation based on the sensitivity analysis and NPV comparison results.

However, several limitations in this study can be improved by further studies. Firstly, this study utilized a time scale of one day for modelling, ignoring the seasonal variation and possible capacity expansion of electricity demand in sewage plants; secondly, the system boundary of this study and the specific implementation process of the DO mechanism have been simplified to a certain extent; finally, the interactions among related stakeholders involved in the supply chain are not considered with regard to the incentive policies design. Future works should verify the proposed model by applying it in real engineering cases and using related experimental data, as well as selecting sewage plants of different sizes and over different operation periods. Furthermore, future studies may adopt game theory to simulate the interactions among stakeholders under policy instruments, and some uncertainty simulation techniques can be integrated for further optimization.

## Appendix

Biogas storage volume 1 (*t*) = Biogas storage volume 1 (*t–dt*)+(Biogas from continues production-Biogas utilization scheme 1)∙*dt*;

Biogas from the first feeding= if TIME<=initial feeding time or TIME>= (initial feeding time+ feeding time interval) then 0 else *V*_max1_∙(1-EXP(−0.06∙ (TIME-initial feeding time)))-*V*_max1_∙ (1-EXP(-0.06∙ (TIME-1-initial feeding time)));

Biogas from the second feeding= if TIME< (initial feeding time+ feeding time interval) then 0 else *V*_max2_∙ (1-EXP(−0.06∙ (TIME-initial feeding time)))-*V*_max2_∙ (1-EXP(−0.06∙ (TIME-1-initial feeding time)))+ *V*_max3_∙(1-EXP(−0.06∙(TIME-initial feeding time-feeding time interval)))-*V*_max3_∙(1-EXP(−0.06∙ (TIME-1-initial feeding time-feeding time interval)));

$$V_{\max i}=\;f\left(\sum X_{n}+\;OLRi\right)$$

*X_n_* = *OLR*_0_∙e^(-*mt*)% where ‘*m*’ denotes the organic degradation rate, and the calculation method for *V*_maxi_ and *X_n_* can be found in Liu et al. (2020);

Biogas demand for flexible production = CH_4_ demand/0.6-Biogas utilization scheme 1

Biogas utilization scheme 1 = Timetable function 1

Biogas utilization scheme 2 = Biogas demand for flexible production/concentration of CH_4_ in biogas;

Concentration of CH_4_ in biogas = Timetable function 2

%the function was cited from the laboratory-scale experiments in Liu et al. (2020);

Real electricity output = Biogas utilization scheme 1∙0.6∙0.4∙10.49+ Biogas utilization scheme 2∙concentration of CH4∙0.4∙10.49;

Electricity demand of sewage plant = Graph(Time)

(0, 300),(1,300),(2, 300),(3.300), (4, 500),(5, 600),(6, 900),(7, 1400),(8, 2100),(9, 2400),(10, 2300),(11, 2400),(12, 2600),(13, 2300),(14, 1900), (15, 1750),(16, 1900),(17, 2100), (18, 2300),(19, 2150),(20, 1800),(21, 1420),(22,900), (23,500);

CH_4_ demand = Electricity demand of sewage plant/(10.49*0.6*0.4);

Revenue from TGC sales = real electricity output∙ TGC price∙13%;

TGC price = Electricity demand of sewage plant*0.00032609−0.097;

Total revenue from TGC sales (*t*) = Total revenue from TGC sales (*t*–*dt*) + Revenue from TGC sales∙*dt*;

Hourly electricity production= 0.75∙MIN (Real electricity output, electricity demand of sewage plant);

Total electricity output (*t*) = Total electricity output (*t*−*dt*) + Hourly electricity production∙*dt*;

Feeding time interval = constant 1;

Initial feeding time = constant 2;

Biogas production rate = Biogas from continues production + Biogas from the first feeding + Biogas from the second feeding

Total biogas production (*t*) = Total biogas production (*t*−1) + Biogas production rate∙*dt*
